# Identification of Novel Lactylation-Related Biomarkers for COPD Diagnosis Through Machine Learning and Experimental Validation

**DOI:** 10.3390/biomedicines13082006

**Published:** 2025-08-18

**Authors:** Chundi Hu, Weiliang Qian, Runling Wei, Gengluan Liu, Qin Jiang, Zhenglong Sun, Hui Li

**Affiliations:** 1 Department of Respiratory and Critical Care Medicine, The Seventh Affiliated Hospital, Sun Yat-sen University, Shenzhen 518107, China; huchundi@sysush.com (C.H.); liugengluan@sysush.com (G.L.); jiangqin@sysush.com (Q.J.); 2 Shenzhen Bay Laboratory, Shenzhen 518000, China; qianweiliang@szbl.ac.cn (W.Q.); rlwei1017@163.com (R.W.); 3 College of Life Sciences, Northwest A&F University, Xianyang 712100, China

**Keywords:** COPD, bioinformatics, biomarker, lactylation, single-cell RNA sequencing, oxidative stress, machine learning

## Abstract

**Objective:** This study aims to identify clinically relevant lactylation-related biomarkers in chronic obstructive pulmonary disease (COPD) and investigate their potential mechanistic roles in COPD pathogenesis. **Methods:** Differentially expressed genes (DEGs) were identified from the GSE21359 dataset, followed by weighted gene co-expression network analysis (WGCNA) to detect COPD-associated modules. Least absolute shrinkage and selection operator (LASSO) regression and support vector machine–recursive feature elimination (SVM–RFE) algorithms were applied to screen lactylation-related biomarkers, with diagnostic performance evaluated through the ROC curve. Candidates were validated in the GSE76925 dataset for expression and diagnostic robustness. Immune cell infiltration patterns were exhibited using EPIC deconvolution. Single-cell transcriptomics (from GSE173896) were processed via the ‘Seurat’ package encompassing quality control, dimensionality reduction, and cell type annotation. Cell-type-specific markers and intercellular communication networks were delineated using the ‘FindAllMarkers’ package and the ‘CellChat’ R package, respectively. In vitro validation was conducted using a cigarette smoke extract (CSE)-induced COPD model. **Results:** Integrated transcriptomic approaches and multi-algorithm screening (LASSO/Boruta/SVM–RFE) revealed carbonyl reductase 1 (CBR1) and peroxiredoxin 1 (PRDX1) as core COPD biomarkers enriched in oxidation–reduction and inflammatory pathways, with high diagnostic accuracy (AUC > 0.85). Immune profiling and scRNA-seq delineated macrophage and cancer-associated fibroblasts (CAFs) infiltration with oxidative-redox transcriptional dominance in COPD. CBR1 was significantly upregulated in T cells, neutrophils, and mast cells; and PRDX1 showed significant upregulation in endothelial, macrophage, and ciliated cells. Experimental validation in CSE-induced models confirmed significant upregulation of both biomarkers via transcription PCR (qRT-PCR) and immunofluorescence. **Conclusions:** CBR1 and PRDX1 are lactylation-associated diagnostic markers, with lactylation-driven redox imbalance implicated in COPD progression.

## 1. Introduction

Chronic obstructive pulmonary disease (COPD), characterized by neutrophilic inflammation and airway remodeling [1,2], represents a leading global cause of mortality with 200 million cases reported in 2019 [3]. Epidemiological studies demonstrate age-dependent prevalence escalation, exerting dual healthcare–economic burdens and comprising life quality metrics [4,5]. Current therapies include bronchodilators, anti-inflammatory agents, and biologics targeting IL-5/CXCR2 pathways, etc. [6,7], and yet molecular pathogenesis and reliable biomarkers remain poorly elucidated.

Histone lactylation, a post-translational modification enriched at regulatory genomic regions, is implicated in malignant and non-malignant diseases [8,9,10,11,12]. Lactate, once viewed only as a metabolic by-product, and its derivative histone lactylation can reshape immune and inflammatory responses, driving disease progression in sepsis and in chronic conditions such as COPD [13,14]. Multi-omics studies show that histone lactylation is widespread across many cell types and is linked to fundamental biological processes [15,16]. In the lung, it regulates macrophage activation and sustains chronic inflammation in COPD and lung cancer [17]. Moreover, lactylation-induced cellular senescence is now considered a key driver of COPD [14,18,19]. However, its diagnostic value in COPD remains unclear, and no lactylation-based biomarkers have yet been identified. Addressing this knowledge gap represents a critical direction for future investigation.

Recent advances in multi-omics technologies have revealed novel perspectives on the molecular mechanisms of COPD. Microarray and bioinformatics analyses have identified several COPD-associated gene modules. For example, Zhong et al. integrated Gene Expression Omnibus (GEO) datasets with the Aging Atlas to uncover the candidate gene hypoxia-inducible factor 1 alpha (HIF1A), which links COPD to aging-related pathways [20]. Gao et al. reported dysregulated genes related to oxidation–reduction imbalance in COPD lung tissue, while Paci et al. applied transcriptomic and network-based approaches to reveal key COPD-related pathways and molecular hubs [21,22]. Despite these advances, the role of post-translational modifications (PTMs), particularly histone lactylation, in COPD remains largely unexplored. However, these studies largely lacked experimental validation and mechanistic depth.

In this study, we employed a machine learning-based integration of microarray and single-cell RNA sequencing (scRNA-seq) datasets from the Gene Expression Omnibus repository to identify histone lysine lactylation biomarkers involved in COPD pathogenesis. scRNA-seq enables the resolution of transcriptional heterogeneity at single-cell resolution, allowing for the identification of cell-type-specific expression programs that are frequently masked in bulk analyses [23,24]. This high-resolution approach is particularly well suited for dissecting histone lactylation-mediated regulatory mechanisms within the complex cellular landscape of COPD.

This study aimed to identify diagnostic molecular targets associated with histone lactylation in COPD. Through single-cell trajectory analysis, we uncovered compartment-specific immune dysregulation, with macrophages emerging as a key affected population. Among the candidate genes identified in our analysis, carbonyl reductase 1 (CBR1) and peroxiredoxin 1 (PRDX1) were selected for experimental validation. Their upregulated expression was confirmed in vitro using BEAS-2B airway epithelial cells via qPCR, immunofluorescence, and Western blotting. Collectively, our findings provide novel insights into the epigenetic landscape of COPD, highlight lactylation-associated biomarker candidates, and lay the groundwork for future diagnostic and therapeutic strategies targeting histone lactylation in chronic lung disease. Given the emerging evidence that lactylation, as a post-translational modification, plays important roles in the pathogenesis of various diseases, we hypothesize that in patients with COPD, lactylated proteins involved in pathological processes such as pulmonary inflammation and oxidative stress may exhibit significant alterations, potentially serving as novel diagnostic biomarkers.

## 2. Materials and Methods

### 2.1. Public Datasets Retrieval

Microarray expression datasets for COPD were retrieved from the GEO database (https://www.ncbi.nlm.nih.gov/geo/, accessed on 18 December 2024) using the R package GEOquery v.2.72.0. The discovery dataset GSE21359, based on the GPL570 platform, includes small airway epithelial samples from 23 COPD patients and 47 healthy controls. The validation dataset GSE76925 contains lung tissues of 111 COPD individuals and 40 non-smoking healthy controls, generated using the GPL10558 platform. To further assess robustness, we additionally used an independent test cohort, GSE38974, consisting of 23 COPD cases and 9 controls (airway/lung tissue specimens). scRNA-seq data from GSE173896, comprising lung tissue from 5 COPD cases and 3 healthy donors [25], were analyzed at single-cell resolution. Informed by prior studies, 332 lactylation-related genes (LRGs) were selected for downstream analysis (Supplementary Table S1) [26]. The overall data processing workflow is illustrated in Figure S1.

### 2.2. Identification and Validation of LRGs Biomarkers

Differentially expressed genes (DEGs) were identified using the ‘limma’ R package v3.60.4 (adj.P.Val < 0.05 and |log2FC| > 0.5) based on the GSE21359 dataset. Weighted gene co-expression network analysis (WGCNA) was applied to the discovery dataset using the ‘WGCNA’ R package v1.72-5 to extract key modules. By integrating overlapping DEGs, LRGs, and WGCNA-derived module genes, candidate biomarkers were obtained. To identify core LRGs, a triad of machine learning approaches—least absolute shrinkage and selection operator (LASSO) regression, Boruta feature selection, and support vector machine–recursive feature elimination (SVM–RFE)—was employed using the ‘glmnet’ package v4.1-8 and the ‘e1071′ R package v1.7-16 with cross-validation. The gene set used for LASSO, Boruta, and SVM–RFE analyses was defined as the intersection of DEGs, WGCNA-derived module genes, and previously reported lactylation-related genes (LRGs), as detailed in Supplementary Tables S1, S3 and S4. Genes selected by all three algorithms were deemed high-confidence biomarkers. Their expression profiles and diagnostic potential were further validated in the GSE76925 dataset using receiver operating characteristic (ROC) curve analysis. The detailed information was available in the Supplementary Materials.

### 2.3. Functional and Regulatory Characterization of Key LRGs

Functional implications of the identified LRGs were explored through protein–protein interaction (PPI) network construction using STRING and ‘igraph’ with an interaction score threshold of 0.4, as recommended in the official STRING documentation [27]. Chromosomal localization was annotated via the ENSEMBL database (https://asia.ensembl.org/index.html, accessed on 25 December 2024) and visualized using ‘Rcircos’ package v1.2.2. To analyze post-transcriptional and transcriptional regulation, integrated miRNA–mRNA and transcription factor (TF)–mRNA networks were constructed. miRNAs were predicted via miRDB (https://mirdb.org/, accessed on 5 January 2025) and miRWalk (http://mirwalk.umm.uni-heidelberg.de/, accessed on 24 January 2025), and TFs were identified using TRRUST (https://www.grnpedia.org/trrust/, accessed on 12 February 2025) and miRNet databases (https://www.mirnet.ca/, accessed on 20 February 2025). Regulatory networks were visualized using Cytoscape 3.10.2.

Pathway enrichment analysis was performed using gene set variation analysis (GSVA) and gene set enrichment analysis (GSEA), based on KEGG gene sets (c2.cp.kegg.v11.0.symbols) from MsigDB (https://www.gsea-msigdb.org/gsea/msigdb, accessed on 15 March 2025). Significantly enriched pathways (FDR < 0.05, |NES| > 1) were visualized using ‘enrichplot’ package v1.24.2. A gene-based nomogram was constructed using the ‘rms’ R package v6.8-2 based on the discovery dataset GSE21359 to evaluate the COPD-related predictive capacity of the validated genes. Calibration plots and decision curve analysis (DCA) were conducted to assess the nomogram’s consistency and net benefit. The overall discriminatory ability of the nomogram was quantified via the area under the ROC curve (AUC). Drug–target associations were investigated through the Drug–Gene Interaction Database (DGIdb) (https://dgidb.org/, accessed on 25 March 2025). Immune microenvironment profiling via the EPIC algorithm and the Wilcoxon rank-sum test identified differentially infiltrated immune cell subsets between COPD and controls.

Single-cell data analysis using ‘Seurat’ package v4.4.0 enabled delineation of cell-type-specific LRG expression and unsupervised clustering. Normalization was performed through the NormalizeData function. Genes showing high variability were detected via the FindVariableFeatures function, employing the variance-stabilizing transformation (VST) approach, selecting the top 2000 genes with the highest variance across cells. The FindNeighbors and FindClusters functions were applied for clustering to delineate distinct cellular populations. To annotate cell clusters, marker genes specific to each cell type were determined through the FindAllMarkers function, configured with the following parameters: logfc.threshold = 0.5, only.pos = TRUE, and min.pct = 0.25. The identification of cell types was informed by the CellMarker resource (http://xteam.xbio.top/CellMarker/, accessed on 19 April 2025) and the prior literature [25]. Differences in abundance between COPD and control groups across various cell types were analyzed using the Wilcoxon rank-sum test. Seurat’s FindMarkers function is used to identify upregulated and downregulated genes in each cell type (COPD vs. control, with thresholds: min.pct = 0.25, logfc.threshold = 0.25), followed by enrichment analysis of the KEGG pathways using the enricher function from the fgsea package v1.30.0. Cell–cell communication dynamics were further explored using ‘CellChat’ at a minimum expression threshold of 0.2, revealing ligand–receptor interaction patterns among major lung cell populations [28]. The above detailed information is available in the Supplementary Materials.

### 2.4. Preparation of Cigarette Smoke Extract (CSE) Preparation and Cell Treatment

CSE was prepared following established protocols [29,30,31], with minor modifications to improve consistency. In brief, smoke generated from a single unfiltered Diamond-brand cigarette (Hebei China Tobacco Industry Co., Ltd., Shijiazhuang, China; tar: 11 mg, nicotine: 1.0 mg, carbon monoxide: 13 mg) was bubbled through 10 mL of serum-free DMEM/F12 medium (Gibco, Thermo Fisher Scientific, Waltham, MA, USA; Cat. No. 11320033) using a modified syringe-driven apparatus over a 5 min period. The resulting extract was filtered through a 0.22 μm membrane to achieve sterilization. Extracts with an optical density difference (ΔOD_320–540_) between 0.9 and 1.2 were considered qualified.

Human bronchial epithelial BEAS-2B cells were provided by Beyotime (Shanghai, China). The cell lines were commercially sourced and originated from normal human bronchial epithelial tissue. The cells were maintained in DMEM/F12 medium, enriched with 10% fetal bovine serum (FBS; Gibco, Cat. No. A5256701) and 1% penicillin–streptomycin (Gibco, Cat. No. 15140122) at 37 °C with 5% CO_2_. Cells were treated with CSE at concentrations determined by viability assays and collected for downstream analysis after a 6 h exposure [32,33].

### 2.5. Experimental Validation

Cell viability was assessed via the CCK-8 assay (Yeasen Biotechnology, Shanghai, China; Cat. No. 40203ES76). Firstly, BEAS-2B cells (1.0 × 10^4^ per well) were seeded into 96-well plates and maintained overnight. On the following day, the cells were treated with different doses of CSE in serum-free medium for 6 h. After adding 10 μL of CCK-8 reagent per well, plates were incubated at 37 °C for 1–2 h, and absorbance at 450 nm was measured.

For gene expression analysis, total RNA was extracted (Vazyme, Nanjing, China; Cat. No. RC102) and reverse-transcribed to cDNA (Vazyme, Cat. No. R433), followed by quantitative PCR (Vazyme, Cat. No. Q712) using GAPDH as an internal control. Relative mRNA expression was calculated using the 2^−ΔΔCt^ method. Supplementary Table S2 contains the primer sequences used in this study.

Immunofluorescence staining was performed in order to assess PRDX1 and CBR1 protein expression. Following 6 h of CSE stimulation, BEAS-2B cells were immersed in 4% paraformaldehyde, blocked with 5% donkey serum (Solarbio, Beijing, China; Cat. No. SL050), and incubated overnight at 4 °C with primary antibodies against PRDX1 (GenuIN Biotech, Cat. No. V61187, China) and CBR1 (GenuIN Biotech, Hangzhou, China; Cat. No. 3044). After washing, cells were treated with Alexa Fluor 488-conjugated secondary antibody and counterstained with DAPI. Fluorescence signals were imaged using a ZEISS LSM980 confocal microscope.

Western blot was performed using total cellular proteins extracted with a commercial lysis kit, followed by BCA quantification. Equal amounts of protein were separated by SDS-PAGE and transferred to PVDF membranes. After blocking with 2% skim milk for 1.5 h at room temperature, membranes were incubated overnight at 4 °C with primary antibodies against PRDX1 (HuaBio, Hangzhou, China; Cat. No. ET1702-08, 1:1000), CBR1 (HuaBio, Cat. No. ER2001-45, 1:1000), and β-actin (HuaBio, Cat. No. HA722023, 1:20,000). HRP-conjugated goat anti-rabbit secondary antibody (Absin, Shanghai, China; Cat. No. abs20040, 1:10,000) was applied for 1 h at room temperature. Signals were developed using an ECL kit (Thermo Fisher Scientific, Waltham, MA, USA; Cat. No. 34577) and visualized with a BioRad imaging system.

### 2.6. Statistical Analysis

GraphPad Prism 10.1.2 was employed to carry out statistical evaluation and generate data visualizations. All experiments were conducted independently and repeated no fewer than three times. Student’s *t*-test was applied for normally distributed data; otherwise, comparisons were performed via the Mann–Whitney U test. A difference was regarded as statistically significant when * *p* < 0.05, ** *p* < 0.01, *** *p* < 0.001, and **** *p* < 0.0001.

## 3. Results

### 3.1. Identification of DEGs and Key Co-Expression Modules in COPD

**Figure 1:**
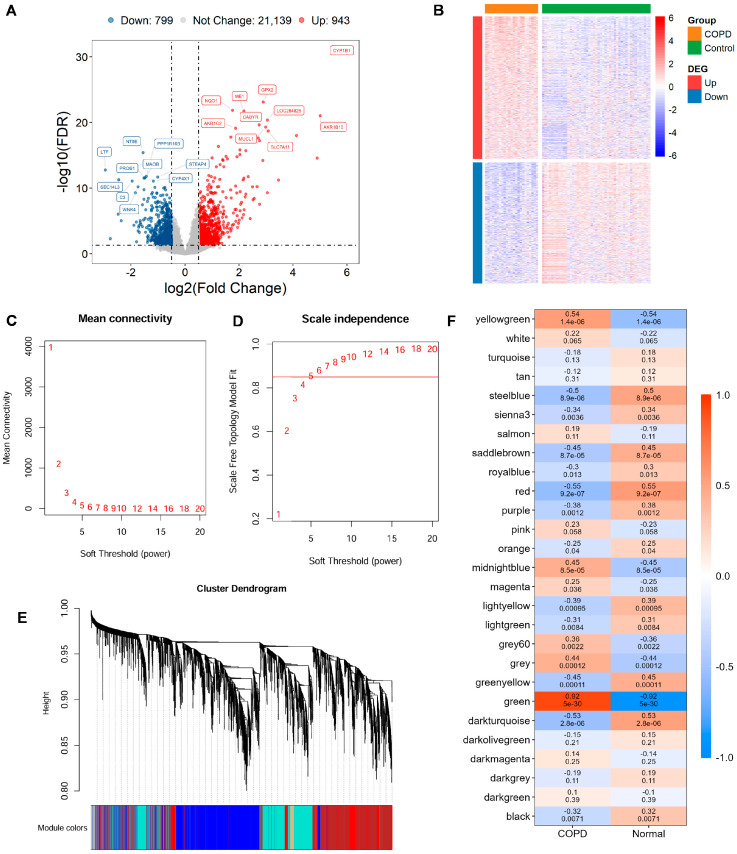
Gene expression differences and module identification by WGCNA. (**A**) Differentially expressed genes (DEGs) are visualized through volcano diagram, with red indicating upregulated genes, sky blue for downregulated genes, and gray for genes showing no significant expression change. (**B**) Expression patterns of significantly altered DEGs in COPD vs. controls are shown in a heatmap, with red indicating high expression and blue indicating low expression. (**C**,**D**) The scale-free topology fit index is assessed across various soft-thresholding powers (β). A soft-thresholding power of 5 is selected, as it first achieves an R^2^ > 0.85, which is considered optimal. (**E**) Original and merged modules are depicted beneath the clustering dendrogram. (**F**) Heatmap of module–trait relationships is presented, where each cell displays both the correlation coefficient and its corresponding *p*-value.

Utilizing the GSE21359 discovery dataset, an in-depth assessment of DEGs was conducted in COPD and normal samples. In total, 1742 DEGs were detected, comprising 943 significantly upregulated genes and 799 with reduced expression in COPD tissues (Figure 1A,B; Supplementary Table S3).

To uncover gene modules closely associated with COPD, WGCNA was conducted on the GSE21359 dataset. Setting the soft power to 5 produced the first scale-free topology fit index exceeding 0.85 and was automatically selected as the optimal parameter (Figure 1C,D). Gene modules were detected through setting the minimum module size at 30 genes, which yielded 38 distinct co-expression modules (Figure 1E). Finally, 18 WGCNA expression modules with significance were identified (Figure 1F). The key green modules showed the most significant correlation with the COPD phenotype (cor = 0.92, *p* = 5 × 10^−30^), highlighting its potential relevance to disease pathology. Therefore, 1952 genes within this module were selected as key candidates for subsequent functional enrichment and mechanistic investigations (Supplementary Table S4).

### 3.2. Identification and Potential Biological Roles of LRG Signatures Within COPD

**Figure 2:**
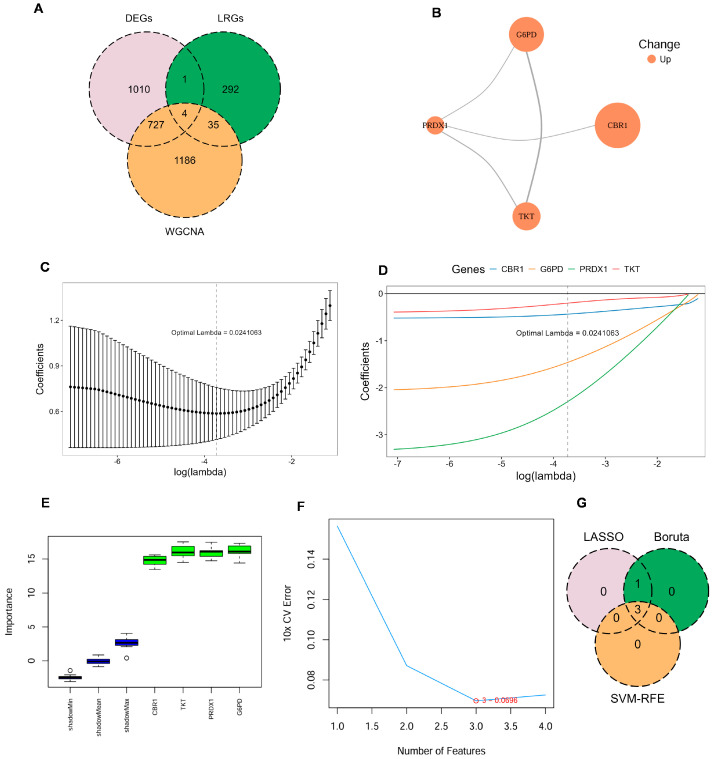
Identification of key LRG signatures in COPD. (**A**) Venn diagram illustrates the overlap between DEGs, genes identified by WGCNA, and LRGs. (**B**) Protein–protein interaction (PPI) network for the candidate genes, constructed using the STRING database, with node color indicating upregulated expression. (**C**,**D**) Ten-fold cross-validation in least absolute shrinkage and selection operator (LASSO) regression model determined the optimal penalty coefficient (λ = 0.024). (**E**) Gene importance scores of candidate genes evaluated by the Boruta algorithm, with green boxes indicating significantly confirmed genes. (**F**) Ten-fold cross-validation error rates under different feature numbers in the support vector machine–recursive feature elimination (SVM–RFE) algorithm, showing the lowest error when the number of features is three. (**G**) Venn diagram illustrating the commonly confirmed key feature genes among the results of the three algorithms.

Four key LRGs were shared among DEGs, genes from the key WGCNA module, and the previously reported LRGs in COPD: Carbonyl Reductase 1 (CBR1), Glucose-6-Phosphate Dehydrogenase (G6PD), Peroxiredoxin 1 (PRDX1), and Transketolase (TKT) (Figure 2A; Supplementary Table S5). To further investigate the potential cooperative functions among these genes, the PPI network revealed clear interactions among CBR1, G6PD, PRDX1, and TKT, forming a tightly interconnected functional module (Figure 2B). These key LRG markers were mainly involved in key oxidation–reduction and metabolic pathways and may represent critical regulators of disease progression.

To further identify LRG signatures in COPD, an integrative machine learning approach was employed based on the GSE21359 discovery dataset, including LASSO regression, SVM–RFE, and Boruta feature selection. In the LASSO regression model, the optimal penalty parameter (λ = 0.024) was determined, under which four genes—CBR1, G6PD, PRDX1, and TKT—were retained as key predictors (Figure 2C,D). Boruta models also identified all four genes as ‘Confirmed’ features (Figure 2E). To further refine the feature set, SVM–RFE analysis revealed that the optimal model was a three-gene model comprising CBR1, G6PD, and PRDX1 (Figure 2F). Therefore, CBR1, G6PD, and PRDX1 were regarded as consensus LRG signatures in COPD (Figure 2G). These LRG signatures across multiple machine learning models further indicated their promise as potential biomarkers for the detection and mechanistic analysis of COPD.

**Figure 3:**
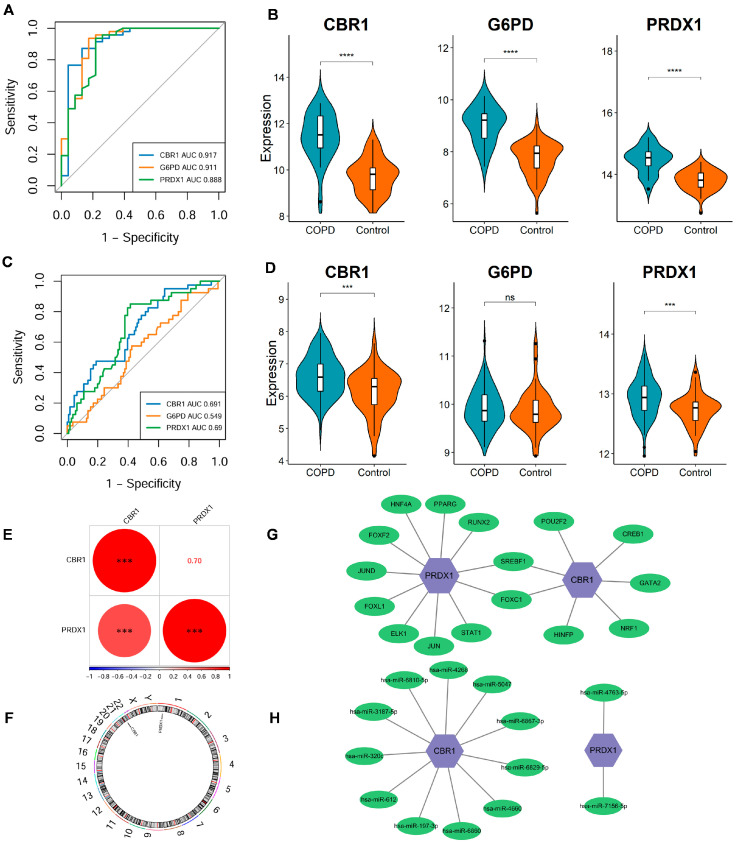
Expression differences, diagnostic performance, and regulatory network analysis of LRG signature genes. (**A**,**B**) ROC curve analysis and expression level comparison in the discovery dataset (GSE21359). **** *p* < 0.0001. (**C**,**D**) ROC curves and expression level analyses in the validation dataset (GSE76925). *** *p* < 0.001; ns, not significant. (**E**) Spearman correlation analysis of CBR1 and PRDX1 expression levels in the discovery dataset. Red and blue circles represent positive and negative correlations, respectively, with size reflecting the coefficient. *** *p* < 0.001. (**F**) Genomic localization of key genes. CBR1 and PRDX1 are located on chromosomes 22 and 1, respectively. (**G**) The transcription factor (TF)–mRNA regulatory network of CBR1 and PRDX1. (**H**) The miRNA–mRNA regulatory network of CBR1 and PRDX1.

The stability and diagnostic value of these signature genes were assessed in the discovery dataset (GSE21359) and an independent validation dataset (GSE76925). In the discovery dataset, ROC curve analysis demonstrated strong diagnostic performance for each gene, with all AUC values exceeding 0.85 (Figure 3A); CBR1, G6PD, and PRDX1 exhibited a significant upregulation in COPD (all *p* < 0.001; Figure 3B). In the external validation dataset, CBR1 and PRDX1 exhibited AUC values > 0.65 as well as significantly elevated expression in COPD patients, supporting their diagnostic robustness across datasets (Figure 3C,D). However, G6PD did not show a significant differential expression and had a qualified AUC of 0.549 (Figure 3C,D), indicating potential variability in its predictive performance across populations.

A significant positive correlation of CBR1 with PRDX1 was identified (r = 0.7, *p* < 0.001), suggesting potential synergistic regulation between these genes (Figure 3E). Additionally, genomic localization analysis revealed that CBR1 and PRDX1 are located on chromosomes 22 and 1, respectively. Despite their different chromosomal positions, these genes may participate in COPD pathology through a shared regulatory network (Figure 3F). Transcriptional regulation analysis revealed that CBR1 is regulated by seven transcription factors, including POU2F2, CREB1, GATA2, and NRF1, which are involved in oxidative stress responses, energy metabolism, and transcriptional regulation (Figure 3G). In contrast, PRDX1 is regulated by eleven transcription factors, including PPARG, RUNX2, STAT1, JUN, and FOXC1, suggesting its pivotal role in antioxidant defense and inflammatory responses (Figure 3G). Post-transcriptional regulation identified eleven potential miRNAs regulating CBR1, including hsa-miR-6810-5p, hsa-miR-5047, and hsa-miR-6829-3p based on the miRTarBase database, indicating that CBR1 may be involved in regulation through miRNA-mediated mechanisms in COPD and other diseases (Figure 3H). PRDX1, in contrast, was predicted to be associated with two miRNAs (hsa-miR-4763-5p and hsa-miR-7156-5p), suggesting that it may also be regulated by specific miRNAs, particularly in oxidative stress or immune regulation processes (Figure 3H). Taken together, these findings establish CBR1 and PRDX1 as reliable lactylation-associated biomarkers for COPD diagnosis and potential development.

### 3.3. Functional Enrichment and Immune Microenvironment Analysis of LRG Signatures in COPD

**Figure 4:**
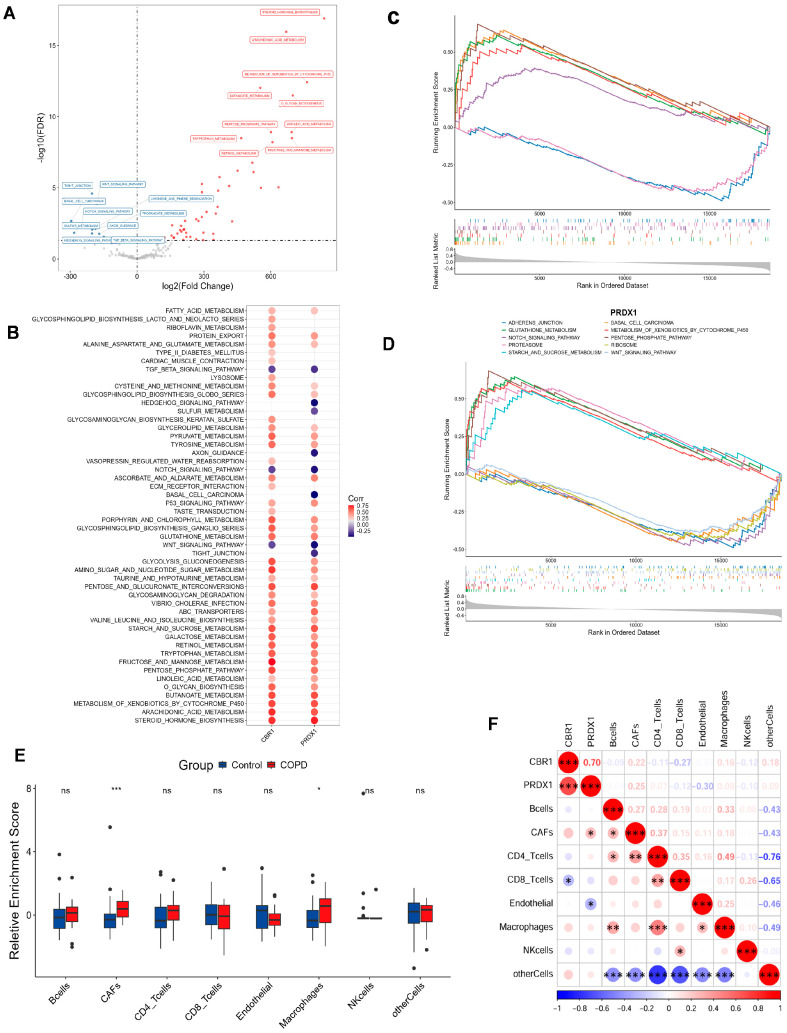
Functional enrichment and immune microenvironment analysis combined with LRG signatures in COPD. (**A**) Volcano plot of differential pathways identified by gene set variation analysis (GSVA) between the COPD and the control groups. Pathways upregulated in the COPD group are shown in red, while those downregulated are shown in blue. (**B**) Correlation analysis between CBR1 and PRDX1 and significantly enriched the KEGG pathways. Circle color indicates the direction of the correlation, with red representing positive and blue indicating negative correlations. Circle size reflects the strength of the correlation. (**C**,**D**) GSEA results for CBR1 and PRDX1 showing the top five significantly enriched signaling pathways for each gene. (**E**) Immune cell infiltration differences in the COPD and the control groups. (**F**) Heatmap of the correlation between gene expression (CBR1 and PRDX1) and immune infiltration, with red indicating positive and blue negative correlations. * *p* < 0.05; ** *p* < 0.01; *** *p* < 0.001; ns, not significant.

To investigate pathway-level differences and explore potential biological mechanisms, the GSVA results identified 60 pathways with significant differential expression between the COPD and the control cohorts (Figure 4A). In comparison with the control cohorts, 50 pathways were notably upregulated in COPD, with the top 5 being metabolism of xenobiotics by cytochrome P450, arachidonic acid metabolism, steroid hormone biosynthesis, butanoate metabolism, and O-glycan biosynthesis. In contrast, 10 pathways were significantly downregulated, with the top 5 being tight junctions, WNT signaling, basal cell carcinoma, Notch signaling, and axon guidance. In addition, CBR1 and PRDX1 were significantly strongly associated with the majority of the differential pathways, including important metabolic pathways such as fructose and mannose metabolism, linoleic acid metabolism, O-glycan biosynthesis, steroid hormone biosynthesis, and metabolism of xenobiotics by cytochrome P450. Additionally, these genes were also positively correlated with tight junctions, Hedgehog signaling, axon guidance, WNT signaling, and TGF-β signaling pathways (Figure 4B). However, no significant correlations were observed for the remaining pathways. Based on these findings, CBR1 and PRDX1 may collaboratively regulate various metabolic and signaling pathways, thus contributing to the pathogenesis of COPD.

To further illuminate the biological functions of CBR1 and PRDX1 in COPD pathogenesis, GSEA was performed and indicated that CBR1 was significantly enriched in several pathways, including O-glycan biosynthesis, metabolism of xenobiotics by cytochrome P450, glutathione metabolism, the pentose phosphate pathway, and oxidative phosphorylation (Figure 4C). In contrast, PRDX1 was primarily enriched in pathways such as glutathione metabolism, the pentose phosphate pathway, metabolism of xenobiotics by cytochrome P450, proteasome activity, and starch and sucrose metabolism (Figure 4D). These results indicated that both genes are likely to have key roles in the pathological mechanisms of COPD through metabolic regulation, oxidation–reduction reactions, and other cellular processes.

In the immune microenvironment analysis, COPD groups showed higher infiltration of cancer-associated fibroblasts (CAFs) and macrophages (*p* < 0.001), suggesting their involvement in the altered immune microenvironment of COPD (Figure 4E). Correlation analysis of the expression levels of CBR1 and PRDX1 with immune cell infiltration revealed that CBR1 was negatively correlated with CD8+ T cell infiltration, while PRDX1 showed a positive correlation with CAFs and a negative correlation with endothelial cells (Figure 4F). These results suggest that CBR1 and PRDX1 may modulate immune cell populations in COPD progression, which provides new theoretical insights for further mechanistic studies and targeted therapies in COPD.

### 3.4. Construction of the Clinical Nomogram Application and Drug Targeting Based on LRG Signatures

**Figure 5:**
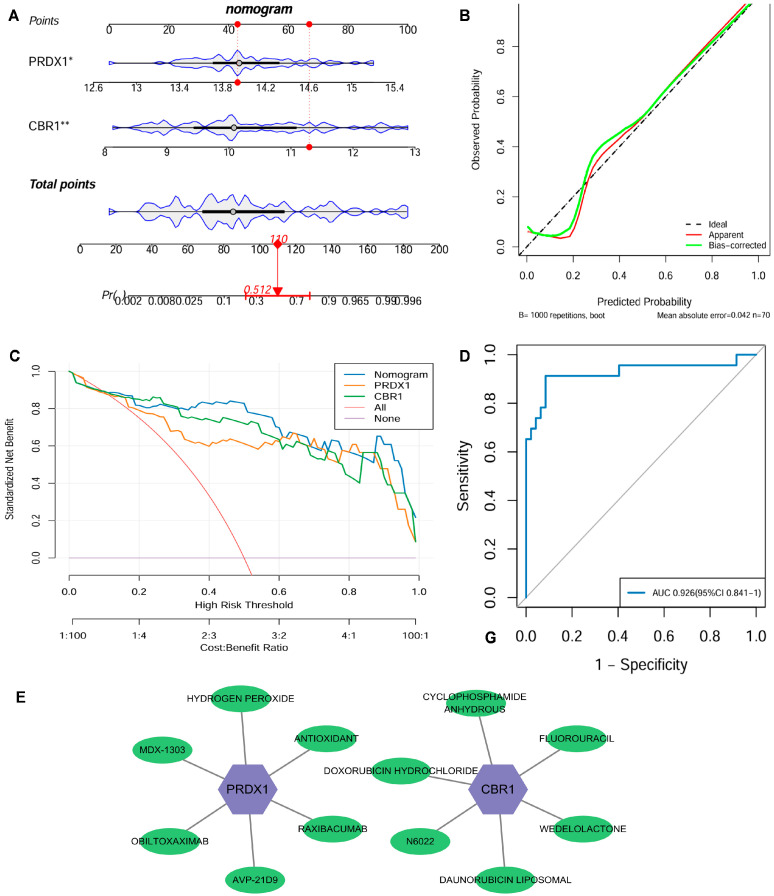
Clinical nomogram and drug screening analysis based on the LRG signatures. (**A**) A nomogram was developed using the expression levels of CBR1 and PRDX1 to evaluate individual risk for the development of COPD. (**B**) Strong agreement was observed between predicted and actual probabilities in the calibration curve. (**C**) Decision curve analysis (DCA) highlighted its clinical benefit over various risk thresholds. (**D**) The nomogram’s receiver operating characteristic (ROC) curve showed an area under the curve (AUC) of 0.926, indicating excellent diagnostic performance. (**E**) A potential drug–gene interaction network related to CBR1 and PRDX1. In the figure, purple hexagonal nodes represent the key genes (CBR1 and PRDX1), while green elliptical nodes indicate the corresponding transcription factors, miRNAs, or drug molecules. * *p* < 0.05; ** *p* < 0.01.

From the GSE21359 discovery dataset of the COPD patients and the control samples, a nomogram integrated with the expression levels of key genes CBR1 and PRDX1 was constructed to quantify their clinical utility in predicting COPD risk (Figure 5A). A calibration curve revealed that ideal curves were closely approximated by the calibration curve, suggesting a strong consistency between the predicted and actual outcomes in this nomogram (Figure 5B). Additionally, the DCA displayed that this nomogram model had a high net benefit over a broad spectrum of risk thresholds, highlighting its significant advantage for clinical application (Figure 5C). The ROC curve further indicated that this nomogram had outstanding diagnostic performance with an AUC of 0.926 (95% CI: 0.841–1.000) (Figure 5D). For the validation cohort GSE76925, we similarly constructed a nomogram using the key genes CBR1 and PRDX1 and generated the corresponding ROC curve, as shown in Figure S2C, which yielded an AUC of 0.705 (95% CI: 0.616–0.794). While this value is lower than the AUC observed in the discovery set, it remains within the range generally considered indicative of moderate predictive performance in clinical biomarker research. Furthermore, an additional independent validation using the GSE38974 dataset (Figure S2D) achieved a high AUC of 0.952 (95% CI: 0.875–1.000), further supporting the robustness of the model. Therefore, the nomogram combined with the LRG signatures (CBR1 and PRDX1) effectively predicts the risk of COPD and exhibits significant advantages in terms of accuracy, stability, and clinical applicability, which holds considerable promise as a tool for early COPD diagnosis and personalized strategies.

At the drug intervention level, potential clinical drug–target interactions were revealed for CBR1 and PRDX1 based on the DGIdb: CBR1 interacts with six drugs, including WEDOLOLACTONE and N6022, suggesting its involvement in anti-inflammatory and antioxidant regulation during COPD pathogenesis; similarly, PRDX1 was found to be associated with six drug molecules, including general antioxidants, further supporting its critical influence in oxidative stress response and immune modulation (Figure 5E). Overall, LRG markers have potential clinical foundation for offering new opportunities for developing therapeutic applications for COPD and related diseases.

### 3.5. Single-Cell Atlas in COPD and Exploration of LRG Signatures at the Single-Cell Scale

**Figure 6:**
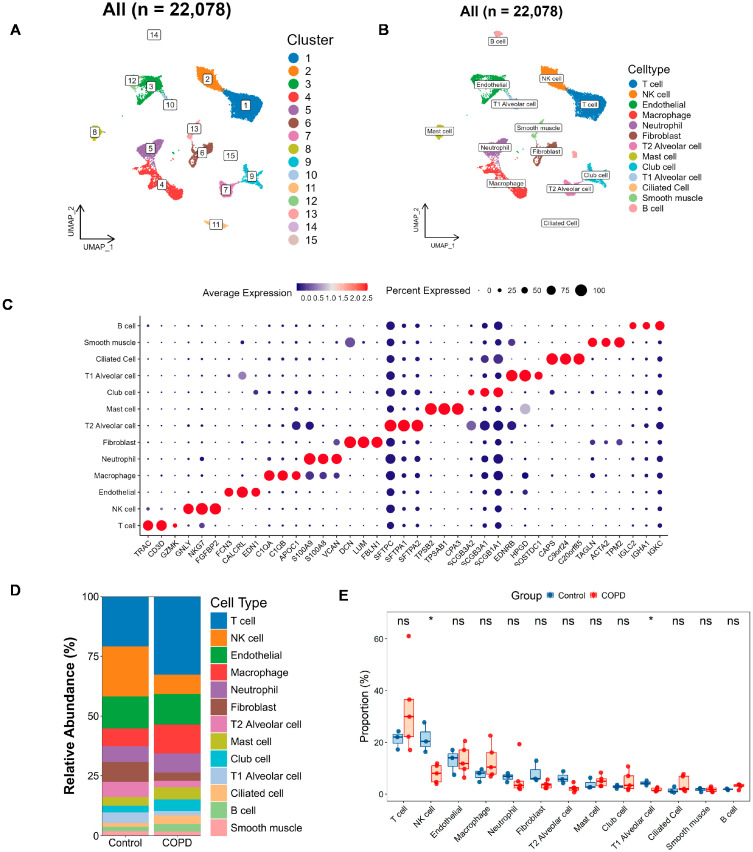
Single-cell atlas and cell–cell communication in COPD. (**A**,**B**) The Uniform Manifold Approximation and Projection (UMAP) clustering (**A**) and annotation (**B**) of 22,078 single cells into 15 clusters and 13 major cell types, respectively. (**C**) Bubble plot depicting marker gene expression, where color reflects average expression and dot size indicates the proportion of cells expressing each gene. (**D**,**E**) Bar and box plots illustrating the relative abundance of each cell type in the COPD and the control groups. The bar plot (**D**) highlights compositional changes across conditions, while the box plot (**E**) presents statistical comparisons of cell-type proportions (two-tailed Wilcoxon test; * *p* < 0.05; ns, not significant).

To investigate key gene levels in COPD at the single-cell level, clustering analysis was performed on a total of 22,078 high-quality cells from 5 COPD and 3 control samples based on the GSE173896 dataset. Dimensionality reduction was performed using UMAP with the 30 PCs, followed by cell clustering, which identified 15 distinct cell populations (Figure 6A and Figure S4A). Annotating these clusters with information from the CellMarker database and from the literature [16], these cells were classified into 13 primary cell types (Figure 6B), including endothelial cells, T cells, NK cells, macrophages, neutrophils, fibroblasts, T2 alveolar cells, mast cells, club cells, T1 alveolar cells, ciliated cells, B cells, and smooth muscle cells. The annotation is focused on the typical marker genes and the top three most significantly expressed markers for each cell subgroup (Figure 6C; Supplementary Table S6).

Next, we plotted bar charts to visualize differences in cellular composition between the two groups (Figure 6D). Preliminary observations revealed that, compared with the controls, the COPD samples contained more T cells, macrophages, and club cells, along with fewer NK cells, fibroblasts, T2 alveolar cells, and T1 alveolar cells. We further validated these differences using rank–sum tests (Figure 6E), which confirmed that NK cells and T1 alveolar cells were significantly reduced in COPD patients.

**Figure 7:**
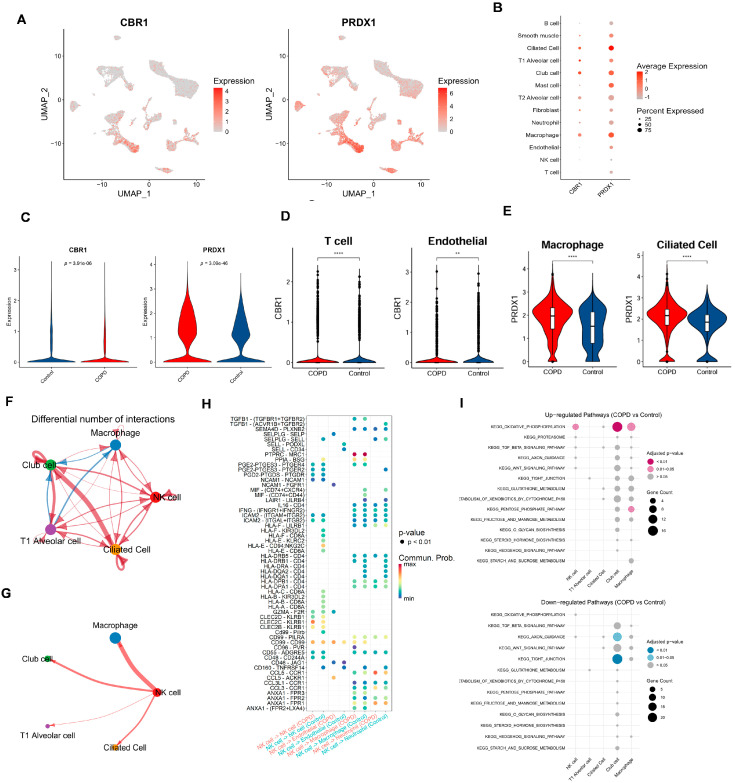
Cell-type-specific expression and intercellular communication features of CBR1 and PRDX1 in COPD. (**A**) UMAPs depicting the single-cell expression profiles of CBR1 and PRDX1 across all cells. (**B**) Bubble plots illustrating average expression and proportion of cells expressing CBR1 and PRDX1 across major cell types. (**C**) Violin plots showing a significantly elevated expression of CBR1 and PRDX1 in COPD compared with the controls (two-tailed Wilcoxon test). (**D**) Cell-type-specific upregulation of CBR1 in T cells and endothelial cells. (**E**) Cell-type-specific upregulation of PRDX1 in macrophages and ciliated epithelial cells. (**F**,**G**) Altered cell–cell communication networks in COPD, highlighting increased interaction frequencies involving macrophages, NK cells, club cells, and ciliated cells. (**H**) Dot plot of differentially regulated ligand–receptor interactions in COPD across key cell types; dot size indicates interaction strength, and color denotes adjusted *p*-value. (**I**) KEGG pathway enrichment analysis of ligand-expressing cells, showing upregulated (top) and downregulated (bottom) pathways in COPD; dot size reflects gene count, and color represents significance. Statistical significance is denoted by ** (*p* < 0. 01) and **** (*p* < 0.0001).

To assess the expression heterogeneity of LRG biomarkers (CBR1 and PRDX1) across different cell types, we generated FeaturePlots to visualize their single-cell expression distributions (Figure 7A). We also constructed bubble plots to display the average relative expression levels of these genes across individual cell types (Figure 7B). Interestingly, these LRG biomarkers were primarily expressed in ciliated cells, T1 alveolar cells, club cells, and macrophages. More specifically, at the overall level, PRDX1 was significantly upregulated in COPD samples, whereas CBR1 showed significant downregulation in the control group (Figure 7C). Further analysis comparing the expression of CBR1 and PRDX1 between groups across each cell type revealed that CBR1 was significantly upregulated in T cells, neutrophils, and mast cells, whereas PRDX1 showed significant upregulation in endothelial cells, macrophages, and ciliated cells (Figure 7D,E and Figure S4B,C). These findings suggest that CBR1 and PRDX1 may contribute to immune regulation and tissue remodeling in COPD through their differential expression across various cell types.

To systematically assess the intercellular communication networks between important cell types, we narrowed the cell–cell communication analysis from all cell types to focus on: (i) cell types with significant abundance differences between groups (NK cells and T1 alveolar cells); and (ii) cell types with a high expression of CBR1 and PRDX1 (ciliated cells, T1 alveolar cells, club cells, and macrophages). Results showed complex differences in intercellular communication between these cell types in COPD vs. controls (Figure 7F–H; Supplementary Figures S4D,E and S5). For example, compared with the controls, macrophages in the COPD samples exhibited weaker communication with club cells but stronger interactions with other cell types. T1 alveolar cells also showed reduced communication with club cells but enhanced interactions with other cell types. Notably, NK cells and ciliated cells in COPD samples displayed stronger interactions with all other cell types (Figure 7F and Figure S4D). In terms of the number and strength of cell–cell interactions, both were lower in the COPD group than in the control group. To provide a comprehensive view of the cell network, communication patterns were further displayed between one specific cell type and four other key cell types in the COPD samples (Figure 7G and Figure S5). Taking NK cells as an example (Figure 7G), NK cells showed the strongest interaction with macrophages, while exhibiting the weakest interaction with T1 alveolar cells. A further comparison of the differences in the interaction intensities of ligand–receptor pairs between NK cells and other cells in the COPD and the control groups (Figure 7H) revealed that there were extensive differences in the interaction intensities of ligand–receptor pairs between different cells between the two groups. These findings reveal the characteristic restructuring of intercellular communication networks between key cell types in COPD, particularly the enhanced signaling interactions among immune-related cells.

Finally, based on the series of functional pathways related to COPD and the LRGs identified in our previous studies, we further sought to examine whether these pathways are significantly enriched in either the COPD or the control group among the five key cell types. Building on our previous identification of functional and metabolic pathways, we selected 15 KEGG pathways involved in metabolism, signaling, cellular structure, and protein degradation, among other biological processes (Figure 7I). Results showed that, compared with the controls, the OXIDATIVE PHOSPHORYLATION pathway was strongly enriched in the NK cells, club cells, and macrophages of the COPD samples—consistent with our previous findings, further supporting the roles of oxidative stress, inflammatory responses, and energy metabolism reprogramming in COPD pathogenesis. Additionally, the club cells in the COPD samples exhibited significant downregulation of the AXON GUIDANCE and TIGHT JUNCTION pathways, suggesting that cell junctions related to the airway epithelial barrier and neurological regulation may be involved in the development and progression of COPD.

### 3.6. CSE Induces Expression of PRDX1 and CBR1 in BEAS-2B Cells

**Figure 8:**
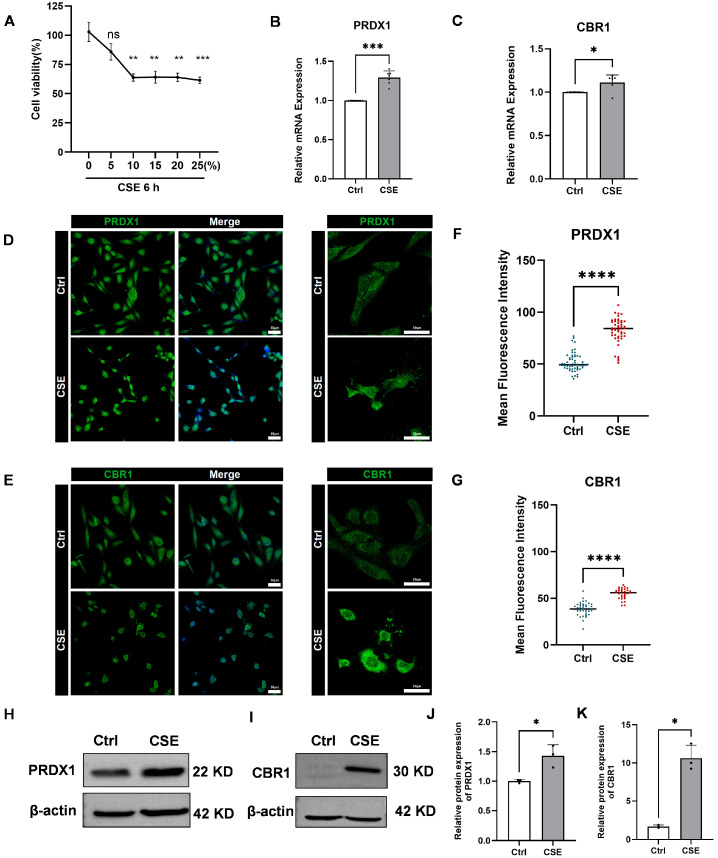
Effects of cigarette smoke extract (CSE) treatment on human bronchial epithelial cells (BEAS-2B), cell viability, and the expression of PRDX1 and CBR1. (**A**) Cell viability of BEAS-2B cells treated with varying concentrations of CSE (0–25%) for 6 h was measured by cell a counting Kit-8 (CCK-8) assay. (**B**,**C**) Quantitative real-time reverse transcription-PCR (qRT-PCR) analysis of PRDX1 (**B**) and CBR1 (**C**) mRNA levels after 6 h treatment with 5% cigarette smoke extract (CSE), using GAPDH as the internal control. (**D**,**E**) Immunofluorescence staining of PRDX1 (**D**) and CBR1 (**E**) showing cellular expression and localization. Left: 20× magnification; right: 40× magnification. (**F**,**G**) Quantification of single-cell fluorescence intensity from 6–10 randomly selected fields per group. (**H**,**I**) Western blot analysis of PRDX1 (**H**) and CBR1 (**I**) protein expression after 6 h CSE exposure. (**J**,**K**) Quantification of relative protein expression levels of PRDX1 (**J**) and CBR1 (**K**), normalized to β-actin. Data are presented as mean ± SEM. Scale bar = 50 μm. * *p* < 0.05; ** *p* < 0.01; *** *p* < 0.001, **** *p* < 0.0001; ns, not significant vs. control (Ctrl).

To explore the toxic effects of CSE on human bronchial epithelial BEAS-2B cells and its impact on the expression of key genes, we first assessed changes in cell viability after 6 h of exposure to different concentrations of CSE using the CCK-8 assay. The results indicated a dose-dependent increase in CSE toxicity, with significant reductions in cell viability observed at concentrations exceeding 10% (Figure 8A). Considering both cell survival rates and physiological relevance, 5% CSE was selected for subsequent experiments, as it simulates mild to moderate oxidative stress damage induced by smoking, while avoiding interference from acute necrosis caused by higher concentrations.

Subsequently, qRT-PCR analysis showed pronounced upregulation of PRDX1 and CBR1 mRNA in BEAS-2B cells after 6 h of 5% CSE treatment (Figure 8B,C). Immunofluorescence staining further validated these findings, revealing increased fluorescence intensity of PRDX1 and CBR1 proteins following CSE exposure (Figure 8D–G). To further validate these findings at the protein level, Western blot analysis was performed, revealing a markedly elevated expression of both PRDX1 and CBR1 in CSE-treated BEAS-2B cells compared with the controls (Figure 8H–K). These results were in strong concordance with the qRT-PCR and immunofluorescence data, collectively confirming that CSE exposure induces robust upregulation of PRDX1 and CBR1 at both the transcriptional and translational levels under oxidative stress conditions. Notably, PRDX1, a crucial thioredoxin peroxidase, is likely upregulated to mitigate hydrogen peroxide induced by CSE and maintain intracellular oxidation–reduction balance. The synchronized upregulation of CBR1 suggests the activation of the NADPH-dependent aldehyde reductase system, potentially involved in the metabolic clearance of lipid peroxidation products (such as 4-HNE), thus providing cellular protection under oxidative stress conditions. Therefore, this study successfully demonstrates that CSE significantly activates the antioxidant defense system mediated by PRDX1 and CBR1, revealing compensatory protective mechanisms initiated in airway epithelial cells during smoking-induced oxidative stress.

## 4. Discussion

The pathogenesis of COPD is complex and primarily involves inflammatory cells and oxidative stress [34]. Various classical and non-classical pathways contribute to the excessive expression of pro-inflammatory factors, contributing significantly to COPD development and chronic lung inflammation [35]. Although the exact pathogenesis of COPD remains unclear, lactylation has emerged as a key player in a variety of acute and chronic pulmonary inflammatory diseases, representing an exciting area of research [36]. However, current studies focus primarily on lactylation related to pulmonary fibrosis, with limited research on its role in other inflammatory pulmonary diseases, including COPD [37]. Therefore, firstly identifying novel and effective diagnostic biomarkers for COPD through lactylation modification represents a promising research direction, potentially offering early intervention strategies that could improve clinical outcomes.

In this study, we explore the association between lactylation and COPD, as well as its diagnostic potential, from a bioinformatics perspective using publicly available chip expression profiles and scRNA-seq data. Additionally, in vitro experiments were conducted to validate key genes. Our study integrated COPD transcriptomic data and scRNA-seq data with LRGs and applied a series of bioinformatics methods to investigate the potential mechanisms of LRGs in COPD. LRG signatures were identified by DEGs, WGCNA, LASSO regression, Boruta models, and SVM–RFE. Following assessment through disease diagnostic AUC values and gene expression consistency in internal and external datasets, two key LRGs were identified: CBR1 and PRDX1. CBR1, a member of the short-chain dehydrogenase/reductase (SDR) family, is NADPH-dependent and catalyzes the reduction of various endogenous and exogenous carbonyl compounds, participating in glutathione and glucocorticoid metabolism. Therefore, it may influence the progression of chronic lung inflammation by modulating cellular oxidation–reduction states and stress responses [38,39,40]. PRDX1, a typical sulfur-specific peroxidase, reduces hydrogen peroxide and organic peroxides, playing a crucial role in maintaining cellular oxidation–reduction homeostasis. It also protects cells from oxidative stress and influences various cellular functions like differentiation, proliferation, and immune regulation [41].

Currently, COPD-related nomogram models show variable performance: radiomics-based models achieve AUCs of 0.846–0.888, while biomarker-based models show AUCs ranging from 0.580 to 0.644 [42,43]. Against this backdrop, we developed an integrated gene-based diagnostic model based on CBR1 and PRDX1 and rigorously assessed its performance using ROC analysis, calibration curves, and DCA. The model demonstrated excellent diagnostic accuracy, with an AUC of 0.926 (95% CI: 0.841–1.000), and provided a high net clinical benefit across multiple risk thresholds. The expression of both genes was significantly elevated in COPD patients in both the discovery (GSE21359) and validation (GSE76925) cohorts, showing consistent discriminative power. A nomogram integrating these genes yielded calibration curves closely aligned with the ideal, further underscoring the model’s clinical applicability.

Moreover, our in vitro model provided robust biological support for these results. After CSE treatment, CBR1 and PRDX1 mRNA and protein levels were notably upregulated in BEAS-2B cells, suggesting that these genes may exert protective effects by modulating oxidative stress responses and metabolic clearance mechanisms. Previous studies have indirectly corroborated the association of these genes with COPD. As an example, Kalabus et al. observed higher CBR1 expression in lung tissues of smokers and lung cancer cells compared with non-smokers [44]. Similarly, Pastor et al. reported that PRDX1 expression was elevated in the bronchoalveolar lavage fluid of COPD patients, distinguishing it from the lung cancer group [45]. These observations support our conclusions, further validating the reliability and clinical potential of CBR1 and PRDX1 as diagnostic biomarkers for COPD.

In our study, CBR1 and PRDX1 demonstrated stable diagnostic performance. To better elucidate their potential mechanisms in the pathogenesis of COPD and explore their association with lactylation, GSEA showed that the two genes are predominantly related to glutathione metabolism, cytochrome P450 metabolism of exogenous compounds, and the pentose phosphate pathway. Prior research has stressed the central importance of glutathione metabolism and the pentose phosphate pathway in maintaining cellular antioxidant capacity [46]. Cytochrome P450 enzymes catalyze a wide array of oxidative and reductive reactions, involving diverse substrate specificities, and may be implicated in the detoxification of exogenous toxins such as those from smoking [47]. These findings suggest that CBR1 and PRDX1 may alleviate oxidative damage and inflammatory responses in COPD by regulating oxidative stress and detoxification pathways. Notably, PRDX1 was also enriched in pathways related to the proteasome, which is closely involved in protein degradation and immune regulation [48]. Therefore, we hypothesize that PRDX1 may influence chronic airway inflammation and lung tissue remodeling by modulating oxidative damage and protein homeostasis.

This study systematically compared the immune microenvironment of COPD lungs with that of healthy controls. We found a marked reduction in NK cells and T1 alveolar epithelial cells in COPD, whereas CBR1 and PRDX1 showed differential, cell-type-specific expression, suggesting that these two lactylation-related genes may regulate immune responses and tissue remodeling through cell-specific pathways. Single-cell communication analysis further revealed a pronounced re-wiring of signaling networks in COPD, with intensified interactions among immune cells (T cells, macrophages, neutrophils, and NK cells), underscoring the central role of chronic inflammation and immune over-activation in disease progression. In parallel, the KEGG AXON_GUIDANCE and the KEGG TIGHT_JUNCTION pathways were significantly downregulated in club cells, indicating epithelial barrier dysfunction and aberrant neuro-regulation that may exacerbate structural damage and persistent inflammation [49,50,51]. Previous studies likewise demonstrate that cigarette smoke activates multiple inflammatory cell types [52,53] and triggers a cascade of cytokine, chemokine, and protease release, thereby accelerating COPD progression [47,48]. The rich body of evidence on inflammatory cells’ direct or indirect roles in the pathogenesis of COPD, particularly regarding oxidation–reduction balance and cellular stress, aligns with our findings.

At the level of key gene regulatory networks, several transcription factors and miRNAs were associated with CBR1 and PRDX1. For example, CREB1, which regulates CBR1, has been extensively studied and is closely linked to oxidative stress responses, mitochondrial function, and chronic pulmonary diseases [54]. On the other hand, STAT1, which regulates PRDX1, is essential for the inflammatory response in COPD [55]. At the miRNA level, our analysis revealed potential regulatory relationships between CBR1, PRDX1, and various miRNAs. Although most of these miRNAs have not been directly associated with COPD, previous studies suggest that several miRNAs, such as miR-21, are aberrantly expressed in pulmonary diseases like COPD and are involved in oxidative stress, macrophage polarization, and lung tissue repair processes [56]. This implies that certain miRNAs within the regulatory network of PRDX1 and CBR1 may also be functional molecules in COPD. Furthermore, existing research has demonstrated that lactate, not only as a metabolic by-product, also functions as an oxidation–reduction signaling molecule between cells and tissues, thereby regulating cellular metabolic states, immune cell activity, and inflammatory responses [12,57,58,59]. In this context, we hypothesize that PRDX1 and CBR1, as typical oxidation–reduction-related enzymes, might participate in regulating cellular oxidative stress, sensing, or influencing lactate-related signaling pathways, thereby contributing to the chronic inflammation and immune microenvironment regulation in COPD. This potential mechanism warrants further validation and exploration in future studies.

This work gives preliminary understanding of the diagnostic value and functional mechanisms of CBR1 and PRDX1 in COPD through multi-omics data analysis and in vitro experiments, but there are still some limitations. First, the limited sample size in the validation dataset may restrict the generalizability of the model’s performance across broader COPD populations. Second, this study did not include sequencing and validation from an in-house cohort, relying instead on publicly accessible databases, which might lead to sample bias and data heterogeneity. Additionally, in vivo animal experiments and more in-depth functional validation, such as gene intervention (knockout/overexpression) and key pathway verification, have not yet been performed. These aspects will be prioritized in our next phase of research to gain a deeper understanding of CBR1 and PRDX1 in COPD pathogenesis and evaluate their potential for clinical diagnosis and treatment.

## 5. Conclusions

This research provides compelling evidence that CBR1 and PRDX1, as key genes associated with lactylation modification in COPD, hold significant potential as biomarkers. Our findings suggest that oxidation–reduction stress associated with lactylation in immune cells may be a key driver of COPD development, introducing a novel perspective on how lactylation modification influences COPD development.

## Data Availability

Data derived from public domain resources: The data presented in this study are available in the Gene Expression Omnibus (GEO) database (https://www.ncbi.nlm.nih.gov/geo/). These data were derived from the following resources available in the public domain: GSE21359, https://www.ncbi.nlm.nih.gov/geo/query/acc.cgi?acc=GSE21359, GSE76925, https://www.ncbi.nlm.nih.gov/geo/query/acc.cgi?acc=GSE76925, GSE38974, https://www.ncbi.nlm.nih.gov/geo/query/acc.cgi?acc=GSE38974, GSE173896, https://www.ncbi.nlm.nih.gov/geo/query/acc.cgi?acc=GSE173896 (all the links were accessed on 31 October 2025).

## Supplementary Materials

The following supporting information can be downloaded at: https://www.mdpi.com/article/10.3390/biomedicines13082006/s1, Figure S1: Flowchart outlining the steps of the analysis; Figure S2: Model validation and generalizability assessment; Figure S3: Quality control, variable gene selection, and principal component analysis of single-cell transcriptomic data; Figure S4: Cell-type-specific expression and intercellular communication alterations of CBR1 and PRDX1 in COPD; Figure S5: Cell-cell communication networks of key signaling source populations in COPD lungs; Table S1: Lactylation related genes from previous research; Table S2: Real-time PCR primers; Table S3: 1742 differential genes; Table S4: Each module gene of WGCNA; Table S5: Intersection of 1742 DEGs, 1952 WGCNA key module genes, and 332 lactylation-related genes, resulting in four disease-related candidates; Table S6: Marker gene expression in 13 cell types from single-cell analysis.
