# Supplementary material for: Identification of Novel Lactylation-Related Biomarkers for COPD Diagnosis Through Machine Learning and Experimental Validation
Source: Biomedicines. 2025 Aug 18;13(8):2006. doi: 10.3390/biomedicines13082006 (PMC12383316; doi:10.3390/biomedicines13082006)
Supplement: Supplementary file 1 [file biomedicines-13-02006-s001.zip › biomedicines-3705010-Supplementary Figures.pdf]

Supplementary Figures

Figure S1.

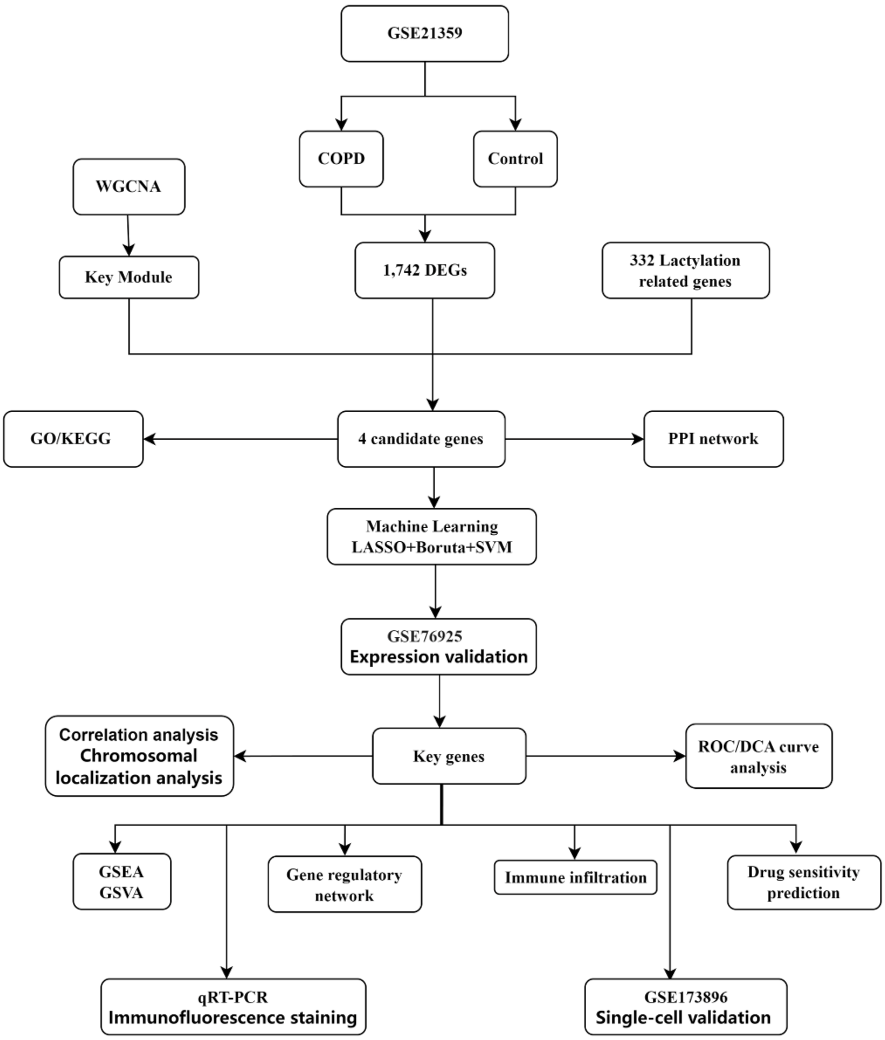

Figure S1. Flowchart outlining the steps of the analysis.

Figure S2.

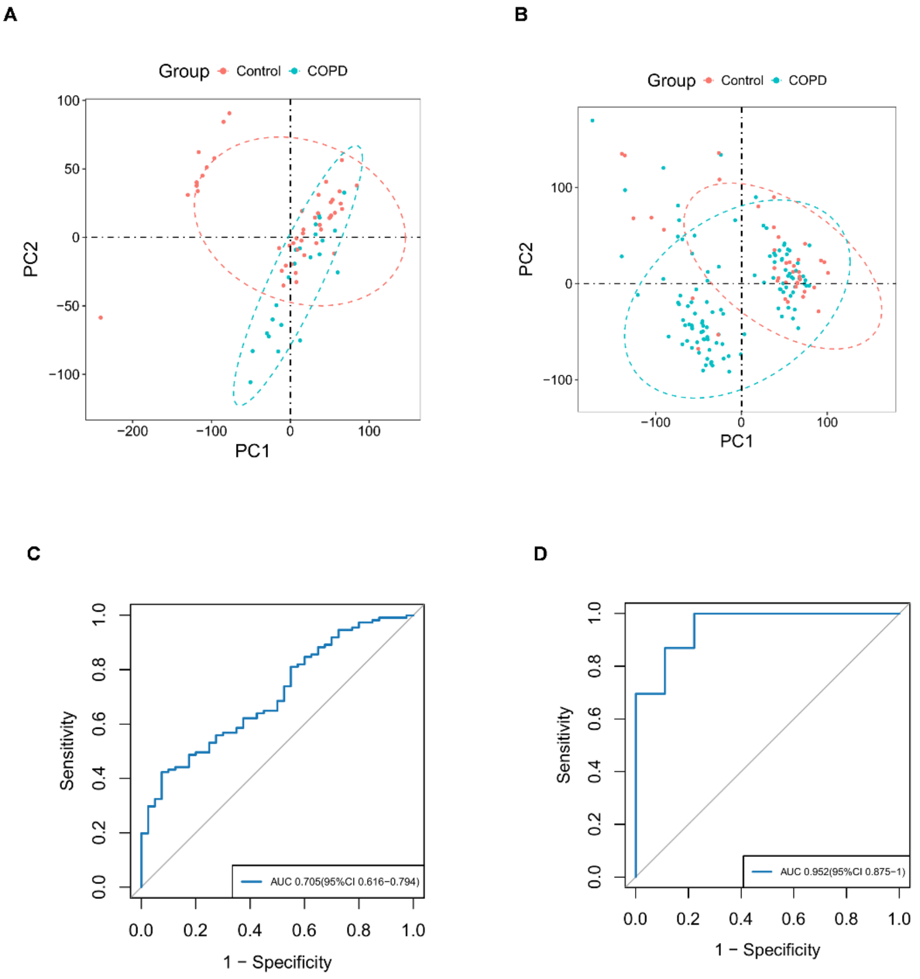

Figure S2. Model validation and generalizability assessment. (A, B) PCA plots of the discovery dataset (GSE21359, A) and validation dataset (GSE76925, B), showing tighter COPD-control separation in the discovery set and greater heterogeneity in the validation set; (C) ROC curve of the nomogram model applied to the GSE76925 dataset, yielding an AUC of 0.705 (95% CI: 0.616-0.794). (D) ROC curve of the nomogram model applied to the independent validation dataset GSE38974, yielding an AUC of 0.952 (95% CI: 0.875 − 1.000).

Figure S3.

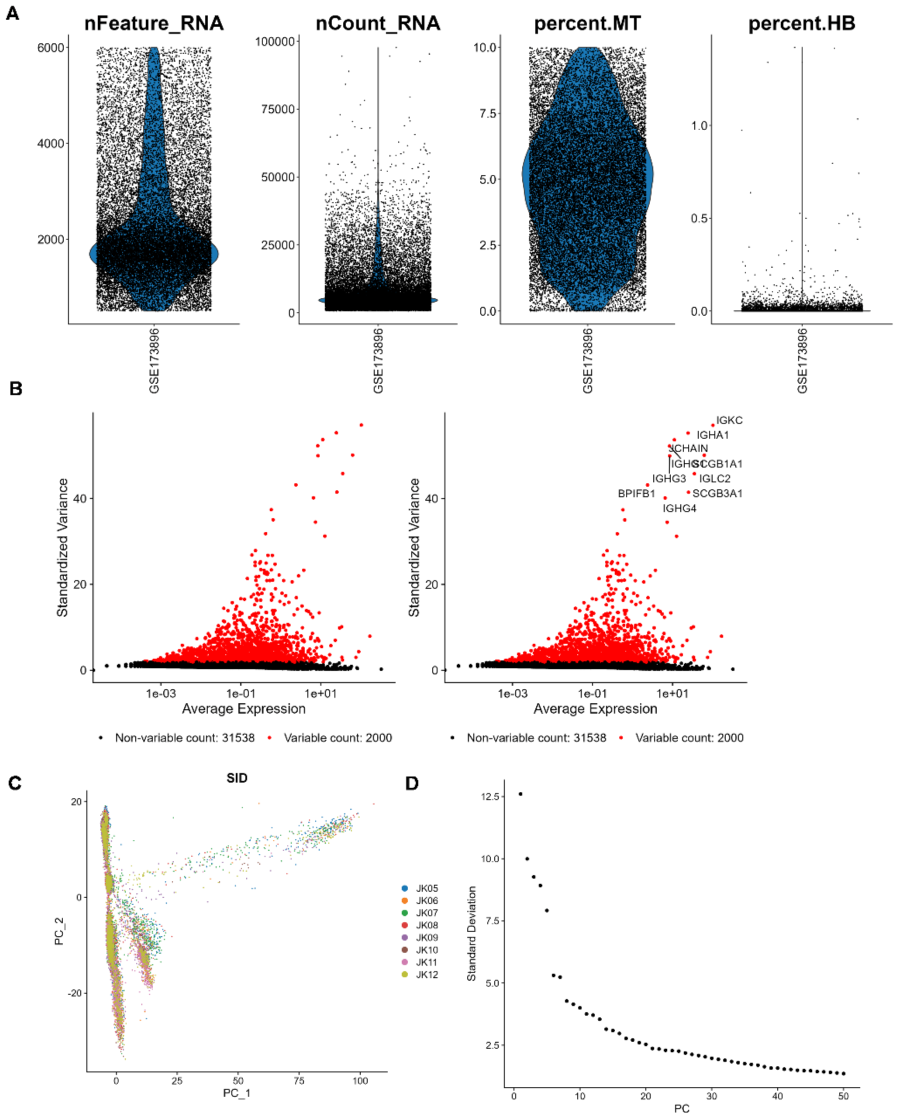

Figure S3. Quality control, variable gene selection, and principal component analysis of single-cell transcriptomic data. (A) Distribution of quality control metrics, including the number of genes per cell (nFeature_RNA), total transcript counts (nCount_RNA), percentage of mitochondrial genes (percent.MT), and percentage of hemoglobin genes (percent.HB); (B) Selection of highly variable genes. Each dot represents a gene, with average expression on the X-axis and standardized variance on the Y-axis. The 2,000 genes with the highest variability are shown in red. The right panel highlights several representative highly variable genes; (C) Principal component analysis (PCA) showing cell distribution across samples. Colors indicate sample origin; (D) Elbow plot showing the standard deviations of the principal components. The top 30 principal components were used for downstream clustering and UMAP dimensionality reduction.

Figure S4.

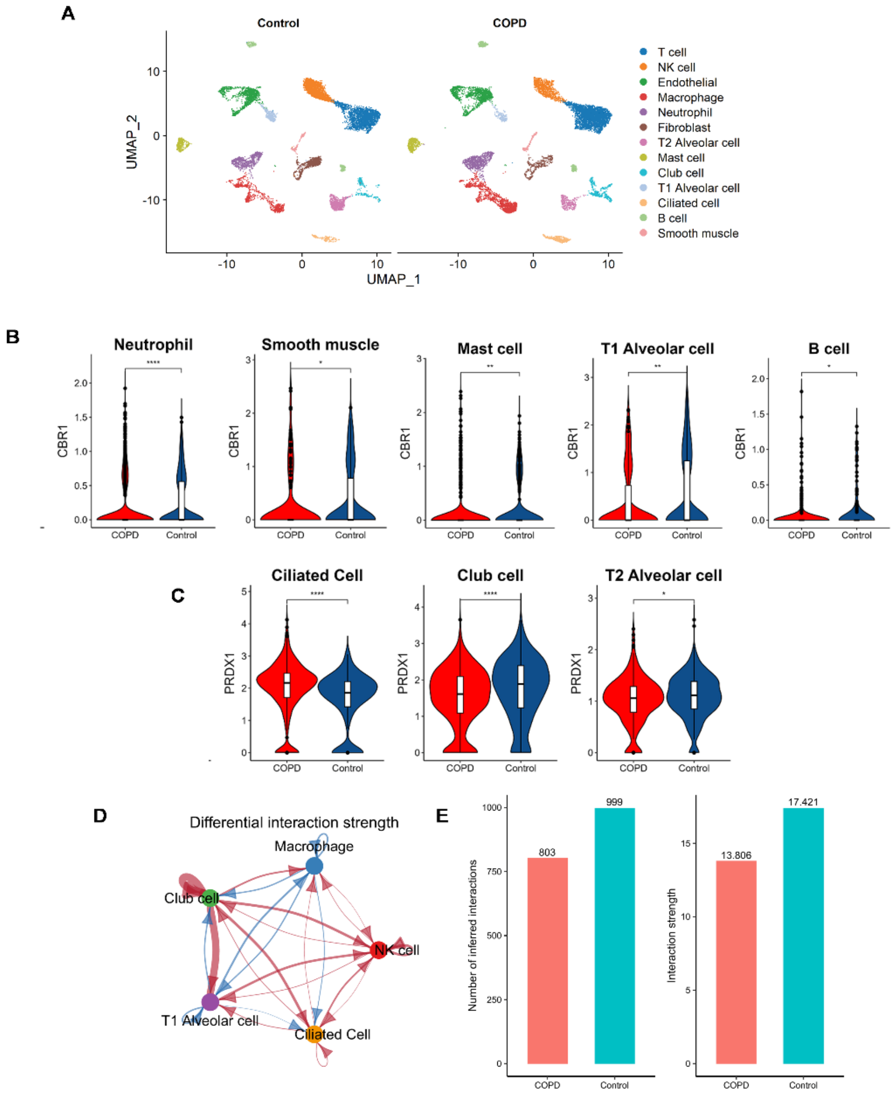

Figure S4. Cell-type-specific expression and intercellular communication alterations of CBR1 and PRDX1 in COPD. (A) UMAP visualization of 13 major lung cell types in control and COPD groups; (B, C) Violin plots showing differential expression of CBR1 (B) and PRDX1 (C) across representative cell types between COPD and control samples; (D) Differential intercellular communication network highlighting changes in interaction strength across key cell types, including macrophages, NK cells, ciliated cells, and club cells; (E) Bar plots comparing the total number and cumulative strength of inferred cell-cell interactions between COPD and control lungs, suggesting impaired intercellular communication in COPD. ****P < 0.0001; ***P < 0.001; **P < 0.01; *P < 0.05; ns, not significant.

Figure S5.

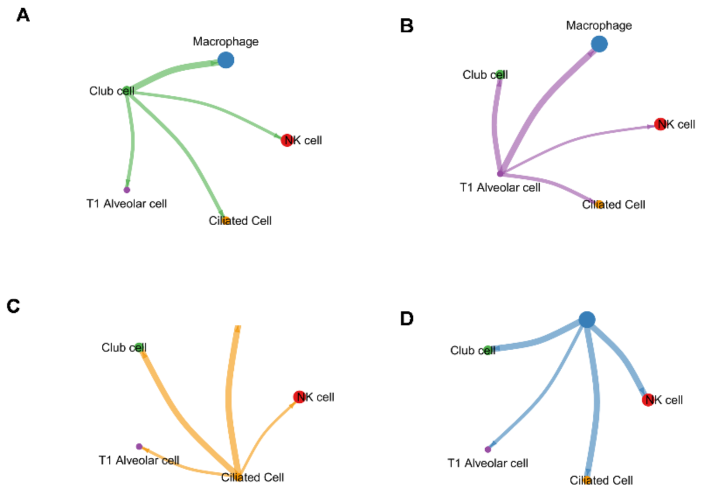

Figure S5. Cell-cell communication networks of key signaling source populations in COPD lungs. (A-D) Outgoing ligand-receptor signaling networks of macrophages (A), T1 alveolar cells (B), club cells (C), and ciliated cells (D) in the COPD group. Arrows indicate the direction of signal transmission to target cell types, with edge thickness representing the strength of the interaction and node size denoting the total signaling output of each source cell type.
