# Supplementary material for: Identification of Novel Lactylation-Related Biomarkers for COPD Diagnosis Through Machine Learning and Experimental Validation
Source: Biomedicines. 2025 Aug 18;13(8):2006. doi: 10.3390/biomedicines13082006 (PMC12383316; doi:10.3390/biomedicines13082006)
Supplement: Supplementary file 1 [file biomedicines-13-02006-s001.zip › biomedicines-3705010-supplementary.pdf]

Supplementary Material

Detailed Methods

Assessment of key markers by differential gene expression and weighted gene co-expression network analyses (WGCNA)

Differentially expressed genes (DEGs) in COPD compared with control samples were determined using the ‘limma’ R package. Genes were regarded as significantly differentially expressed if they exhibited the adjusted p-value (adj.P.Val) < 0.05 and absolute log2 fold change (|log2FC|) > 0.5. A volcano and heatmap graph were produced to display the DEGs.

The WGCNA was further performed to explore expression pattern networks across samples for the identification of COPD-related gene modules. The ‘WGCNA’ R package was used to execute the ‘goodSamplesGenes’ procedure, enabling hierarchical clustering of the training dataset and to detect outliers, which were subsequently removed (Figure S1A). An adjacency matrix was constructed by calculating pairwise Pearson correlation coefficients between genes. A soft-thresholding power $\beta = 10$ (scale-free topology fit index $R^2 = 0.85$) was selected to ensure the construction of a scale-free network. Topological overlap measures were calculated to identify functional modules, and gene modules with their module eigengenes (MEs) were defined using a dynamic tree-cutting algorithm (Figure S1B). To identify trait-associated modules, module-trait correlation matrices were computed by correlating MEs with COPD phenotypic traits. Modules showing an absolute correlation coefficient > 0.5 and a p-

value < 0.01 were deemed key modules. Genes within these modules were subjected to further analysis to identify candidate targets relevant to COPD pathogenesis. The overlap genes were shown with the R package ‘Venn’ among DEGs, WGCNA-derived module genes, and LRGs, enabling the identification of key lactylation-related candidate genes and selection for downstream analyses.

Machine learning-based identification of core genes

In order to optimize the selection of candidate genes associated with lactylation and determine the most informative biomarkers, we utilized a combination of three machine learning techniques: least absolute shrinkage and selection operator (LASSO), Boruta, and support vector machine-recursive feature elimination (SVM-RFE). Integrating these three algorithms leverages their respective strengths, enhancing diagnostic model accuracy, interpretability, and stability while minimizing the risk of overfitting. Using the gene expression profiles of candidate genes from the training dataset GSE21359 as input features and group labels (COPD vs. control) as outcome variables, each of the three algorithms was independently applied to identify the most critical genes for downstream analysis. First, utilizing the ‘glmnet’ R package, a LASSO regression model was developed. The optimal penalty parameter was determined through ten-fold cross-validation. Next, the significance of the selected genes was assessed using the Boruta algorithm. By introducing shadow attributes as random baselines and employing Mann-Whitney U tests to compare Z-score distributions, Boruta identified significant genes as ‘Confirmed’ features. To further refine the feature set, SVM-RFE analysis was

conducted utilizing the ‘e107’ R package, with 10-fold cross-validation. This iterative algorithm eliminated less informative features while minimizing prediction error. And the optimal model was selected based on the lowest cross-validated error. A Venn diagram analysis integrated the outputs of all three algorithms as consensus signature genes.

Efficiency evaluation, correlation, and regulatory networks of key LRG biomarkers

To assess the stability and classification capability of the core genes identified through machine learning, an independent external dataset (GSE76925) was introduced as a validation cohort. The expression profiles of these candidate genes were analyzed and compared between individuals with COPD and healthy controls. To evaluate the diagnostic effectiveness of each gene independently, receiver operating characteristic (ROC) curves were constructed using the ‘pROC’ R package. Genes exhibiting statistically significant differential expression ($p < 0.05$) in the training as well as validation datasets were incorporated into the final diagnostic model.

A protein-protein interaction (PPI) network was constructed to investigate potential protein-level interactions by submitting the selected candidate genes to the STRING database (https://string-db.org/), with a minimum interaction score threshold of 0.4. The network was subsequently exported and visualized using the ‘igraph’ R package to depict the interaction landscape and expression changes among key genes. Spearman

rank correlation analysis was applied to investigate potential interrelationships among the key genes utilizing the 'stats' R package, and a correlation heatmap was generated to visualize gene-gene associations. Additionally, to map the chromosomal locations of the key genes, genomic position information was acquired through the ENSEMBL database (https://asia.ensembl.org/index.html). The 'Rcircos' R package was then used to annotate and visualize the spatial distribution of key genes across human chromosomes.

To investigate the complexity and diversity of regulatory mechanisms underlying the expression of key genes, the integrated miRNA-mRNA and transcription factor (TF)-mRNA regulatory networks were constructed. Putative miRNAs targeting the key genes were predicted using two independent databases, miRDB (https://mirdb.org/) and miRWalk (http://mirwalk.umm.uni-heidelberg.de/). The overlapping miRNAs predicted by both databases were defined as key miRNAs. The interaction network between miRNAs and target genes was graphically represented using Cytoscape. TFs potentially regulating the key genes and predictions were identified via miRNet (https://www.mirnet.ca/) using data from the TRRUST database (https://www.grnpedia.org/trrust/). A TF–mRNA regulatory network was built and graphically rendered with the aid of Cytoscape, alongside the miRNA-mRNA network.

Construction and evaluation of clinical nomogram and drug selection

For the purpose of evaluating the COPD-related predictive capacity of the validated genes, a gene-based nomogram was constructed using the 'rms' R package, based on the training dataset GSE21359. The constructed nomogram quantifies the contribution of each gene by assigning a specific score based on its expression level. These individual scores are subsequently aggregated to calculate an overall score, which reflects the estimated likelihood of developing COPD. Calibration plots generated via the 'rms' package assessed the consistency linking predicted to actual clinical outcomes; a calibration curve slope close to 1 indicates high predictive accuracy. To determine the potential clinical applicability of the predictive model, decision curve analysis (DCA) was conducted utilizing the 'rmda' package; a net benefit greater than zero suggests a favorable predictive effect. The overall discriminatory ability of the nomogram was further quantified via the area under the ROC curve (AUC). In order to explore clinical candidate compounds with potential therapeutic relevance to the identified core genes, drug–target associations were investigated through the Drug–Gene Interaction Database (DGIdb) (https://dgidb.org/).

Functional enrichment analysis based on gene set variation analysis (GSVA) and gene set enrichment analysis (GSEA)

To explore pathway-level differences between COPD and control samples, we performed GSVA on the training cohort using the 'clusterProfiler' R package. For each KEGG signaling pathway, enrichment scores were derived using the curated gene set file 'c2.cp.kegg.v7.4.symbols.gmt', which was accessed via the Molecular Signatures

Database (MsigDB; https://www.gsea-msigdb.org/gsea/msigdb). The 'limma' package was used to assess variations in pathway activity between the COPD and the control groups, applying a significance cutoff of $p < 0.05$. Correlation analysis between key gene expression levels and the activity of significantly enriched pathways was performed via the rcorr function provided in the 'Hmisc' package, with correlations considered meaningful at $R > 0.3$.

GSEA was performed using the c2.cp.kegg.v11.0.symbols gene set, downloaded from the Molecular Signatures Database (MSigDB), to further elucidate the potential functional roles of the key genes in COPD. The 'stats' R package was employed to conduct Spearman rank correlation in the training cohort, evaluating the association of each key gene with the entire gene set. Genes were sorted from highest to lowest according to their correlation scores for each key gene. These ranked lists were then subjected to GSEA via the 'clusterProfile' R package. Pathways were considered significantly enriched if they met the criteria of $|NES| > 1$ and a false discovery rate (FDR) < 0.05. The 'enrichplot' package was used to visualize the top five most significantly enriched upregulated and downregulated KEGG pathways.

Immune microenvironment analysis

To evaluate immune cell infiltration in COPD versus control samples, the EPIC algorithm in the 'IOBR' function was used to determine the relative abundance for immune-related cell populations based on the training cohort. Eight major immune cell

types were compared between the two groups, including only those with $p < 0.05$ and non-zero inter-sample standard deviation. Stacked bar charts depicting the relative distribution of immune cell populations across different samples were created using the 'ggplot2' visualization package. Immune cell infiltration levels between COPD patients and healthy controls were compared using the Wilcoxon rank-sum test, with immune cell types showing statistically significant differences being defined as differentially infiltrated immune cells.

scRNA-seq data processing

Using the 'Seurat' R package, the scRNA-seq data from GSE173896 were processed and analyzed. Quality control was performed based on the following criteria: (a) genes detected in no fewer than three individual cells were kept; (b) cells with expression levels ranging from 200 to 6000 genes and exhibiting mitochondrial content under 20% were selected; and (c) cells presenting mitochondrial gene proportions < 25% and hemoglobin expression < 3% were retained (Figure S2A). Normalization was performed through the NormalizeData function. Genes showing high variability were detected via the FindVariableFeatures function, employing the variance-stabilizing transformation (VST) approach, selecting the top 2000 genes with the highest variance across cells. The dataset was standardized through the ScaleData function to prepare for dimensionality reduction. Principal component analysis (PCA) was implemented via the RunPCA function, according to the top 2000 variable genes and to the most statistically significant principal components as determined by the JackStrawPlot

function (Figure S2B). A total of 30 principal components were retained for further analysis (Figure S2C-D). The FindNeighbors and FindClusters functions were applied for clustering to delineate distinct cellular populations.

To annotate cell clusters, marker genes specific to each cell type were determined through the FindAllMarkers function, configured with the following parameters: logfc.threshold = 0.5, only.pos = TRUE, and min.pct = 0.25. The identification of cell types was informed by the CellMarker resource (http://xteam.xbio.top/CellMarker/) and prior literature[(1)]. The expression of canonical marker genes across clusters was visualized using bubble plots, and labeled cell populations were embedded into a two-dimensional space through Uniform Manifold Approximation and Projection (UMAP).

Cell-cell communication analysis

Cell-cell communication is a fundamental biological process that enables cells to coordinate responses to internal and external stimuli. To elucidate this process, ligand-receptor interactions were evaluated using the 'CellChat' R package, a powerful framework for analyzing the co-expression of ligand-receptor pairs across cell populations and for decoding intercellular signaling networks[(2)]. To enhance biological relevance and reduce noise from low-expressing genes, a minimum expression threshold of 0.2 was applied to filter ligand-receptor pairs included in the analysis.

Supplemental references

1. Watanabe N., Fujita Y., Nakayama J., Mori Y., Kadota T., et al. (2022) Anomalous Epithelial Variations and Ectopic Inflammatory Response in Chronic Obstructive Pulmonary Disease. American journal of respiratory cell and molecular biology 67, 708-719.

2. Jin S., Guerrero-Juarez C.F., Zhang L., Chang I., Ramos R., et al. (2021) Inference and analysis of cell-cell communication using CellChat. Nature communications 12, 1088.
